# The importance of remembering

**DOI:** 10.7554/eLife.30599

**Published:** 2017-08-14

**Authors:** Eve Marder

**Affiliations:** 1 Volen CenterBrandeis UniversityWalthamUnited States; 2 Biology DepartmentBrandeis UniversityWalthamUnited States

**Keywords:** Living science, memory, teaching

## Abstract

Creativity in science requires the ability to recall information and data, and will suffer if we rely too much on technology to remember things for us.

Much has been written about the importance of remembering the lessons of history. Surely we should be able to recall the mistakes that led us to fascism, or to many of the other deplorable actions of our collective and individual pasts, so that we can use the past to avoid repeating previous errors in politics, social policy, and health care. But, sadly, it appears that human nature benefits less from past mistakes than it ought to, perhaps because painful memories are painful. That said, we recognize that our ability to live our lives depends on our memory and our ability to make informed decisions. We understand that neurological disorders that are accompanied by profound memory loss make it impossible for some people to live and function completely in the world. It is not surprising, therefore, that understanding the cellular, molecular and circuit mechanisms underlying memory and learning is one of the biggest challenges in neuroscience. But it is also interesting that the role of memory in our everyday lives is changing as we move into a technology-dominated universe in which we use phones and other devices to store and access information that we once kept in our heads. Perhaps this is progress, but I am not sure.

Those of us who teach undergraduate and graduate students daily confront the fact that our students often do not remember what they learn in class or read, even if they get almost perfect scores on their exams. Our students have seemingly moved storage from their brains to their phones or tablets. What is more, they are completely convinced that because they can, in principle, access the world’s knowledge with a few key strokes, there is little reason for them to remember what they studied the previous semester, or read last month. I first discovered this when I would query my students if they had studied something in another class the year before, and 80 heads would shake "no". Then I would check with the professor of that class only to discover it had been taught and these same students had answered exam questions on that exact topic!

My students’ failure to remember drives me to distraction. Although I rarely pull rank and give my students orders, I have started telling them that they are not allowed to forget what they studied last year or read last week. Of course I know that they are constantly accompanied by devices that access the cloud and can answer their queries. So why do I care that students in 2017 don’t remember what they have learned? I just cannot get my head around students who don’t remember their own data. (That said, I have known senior investigators who felt it was easier to repeat an experiment than it was to retrieve the results of an experiment done years previously, and I find this equally incomprehensible.)

**Figure 1:**
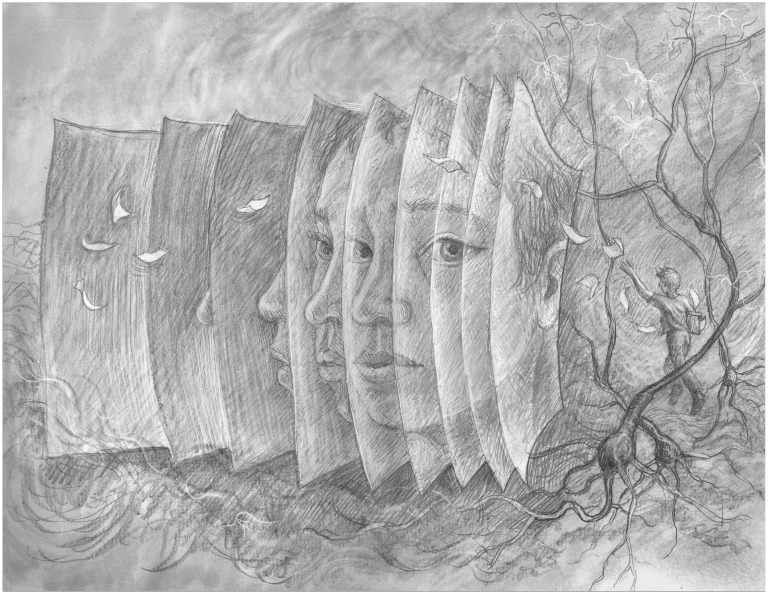
More and more students are forgetting everything they have learned because they can "just look it up", but this will reduce their ability to do creative science.

Illustration: Ben Marder

But the real reason I care so much about the scientist’s brain being an important storage device for experiments done, papers read and courses studied is that it is impossible to think creatively into the future without a sense of what is known. We commonly say that we are looking for interdisciplinary and synthetic thinkers, who can make connections between disparate fields, and see new paths for discovery. I cannot imagine finding those creative leaders for the future among the legions of students who forget everything they have learned because they can "just look it up". How does one know what to look up if one has forgotten so much?

When I was young I had an outstanding memory. At 16, I could remember the names and dates of all of the American Presidents, I knew all of the Amendments to the US constitution, and I learned biochemical pathways effortlessly. By the time I was a 4th year PhD student my memory was no longer as good as it was at 16. So, when I met Ted Bulloch, one of the greats in neuroscience, and I discovered that he seemed to remember every paper he had ever read, I asked how he did it. "You just have to decide to remember," he answered. I still remember how taken aback I was by his statement, because I thought that memory was automatic, and one had little control over it. I know full well that people differ greatly in their ability to remember, and I certainly don’t today have the memory I had when I was 16 or 25. But even so, I suspect that there is a deep message in what Ted said, and that we would all be better scientists if we remembered to remember more of what we learn about science. It is impossible to reason with the forgotten, and every time we give ourselves permission to forget, we are forgoing the opportunity to make new and interesting connections between what has been discovered in the past in seemingly disparate domains of enquiry.

Biology will inevitably, in time, degrade all of our memories, but I suspect that most truly creative scientists take full advantage of the memories they have stored in their brains as they make new discoveries.

